# Optimization of a MOF Blended with Modified Polyimide Membrane for High-Performance Gas Separation

**DOI:** 10.3390/membranes12010034

**Published:** 2021-12-27

**Authors:** Yushu Zhang, Hongge Jia, Qingji Wang, Wenqiang Ma, Guoxing Yang, Shuangping Xu, Shaobin Li, Guiming Su, Yanqing Qu, Mingyu Zhang, Pengfei Jiang

**Affiliations:** 1 Heilongjiang Provinces Key Laboratory of Polymeric Composite Materials, Department of Chemical and Chemical Engineering, Qiqihar University, Wenhua Street, Qiqihar 161006, China; 18204667530@163.com (Y.Z.); mfeather7@163.com (W.M.); qqhrlsb1022@126.com (S.L.); vipquyanqing@163.com (Y.Q.); zhangmingyuno1@163.com (M.Z.); jpf848185@163.com (P.J.); 2 CNPC Reasearch Institute of Safety & Environment Technology, Changping District, Beijing 102249, China; wangqingji@petroChina.com.cn; 3 Synthetic Resin Laboratory, Daqing Petrochemical Research Center, Petrochemical Research Institute, No. 2, Chengxiang Road, Wolitun, Longfeng District, Daqing 163714, China; ygx459@petrochina.com.cn; 4 Institute of Advanced Technology, Heilongjiang Academy of Sciences, No. 52, Renhe Street, Nangang District, Harbin 150009, China; suguim@163.com

**Keywords:** mixed matrix membrane, metal-organic frameworks, polyimide, gas separation

## Abstract

The preparation, characterization and gas separation properties of mixed matrix membranes (MMMs) were obtained from polyimide capped with ionic liquid and blended with metal-organic frameworks (MOFs). The synthesized MOF was amine functionalized to produce UiO-66-NH_2_, and its amino group has a higher affinity for CO_2_. Mixed matrix membranes exhibited good membrane forming ability, heat resistance and mechanical properties. The polyimide membrane exclusively capped by ionic liquid exhibited good permselectivity of 74.1 for CO_2_/CH_4_, which was 6.2 times that of the pure polyimide membrane. It is worth noting that MMM blended with UiO-66-NH_2_ demonstrated the highest ideal selectivity for CO_2_/CH_4_ (95.1) with a CO_2_ permeability of 7.61 Barrer, which is close to the 2008 Robeson upper bound. The addition of UiO-66-NH_2_ and ionic liquid enhanced the permselectivity of MMMs, which may be one of the promising technologies for high performance CO_2_/CH_4_ gas separation.

## 1. Introduction

Concerns about global warming have brought unprecedented public attention on the issue of carbon emissions [1,2,3,4]. As a result, an effective technique for separating the carbon dioxide from a mixture is required. Gas separation membrane technology is an effective method. The objective that researchers have been trying to achieve is a membrane material with high permeability and high selectivity. While gas separation membranes have advanced significantly, numerous gas separation membranes with excellent characteristics have emerged, such as polymer membranes [5]; metal-organic framework membrane (MOF) [6,7,8]; carbon membranes and zeolite membranes [9,10]; and various mixed matrix membranes (MMM) [11,12,13]. Polymer membranes are the most important commercial membranes for gas separation due to their advantages, which include the ease of membrane formation and low cost. However, polymer membranes are usually limited in their equilibrium relationship between selectivity and permeability, which makes it difficult to achieve both simultaneously [14,15]. Robeson applied extensive experimental data to demonstrate the inverse relationship between selectivity and permeability of polymer membranes and defined the Robeson upper bound [16,17]. For the past two decades, the focus of separation membrane research has been on how to exceed the upper bound. Common membrane modification methods include thermal rearrangement modification [18,19]; grafting modification [20]; and mixed matrix membranes, etc.

Metal organic framework materials (MOFs) are a new type of crystalline porous materials with tunable structure and function, which are formed by coordinated self-assembly of metal clusters/ions and ligands [21,22,23]. Metal-organic skeleton materials are widely used in various industries, including as catalysts; liquid phase separation; and hydrogen storage and gas separation due to their high porosity and good chemical stability. However, with industrially produced tunable separation membranes with excellent properties of metal-organic skeletal materials, it is necessary to focus on how to simultaneously produce separation membranes with high selectivity; high permeability; high mechanical strength; and stability. Bernabe et al. [24,25] presented a modified polyimide membrane using microporous aluminum fumarate (A520) as the filler to improve permselectivity for CO_2_/N_2_ and O_2_/N_2_. When compared to the pure PI membrane, these membranes have improved CO_2_ permeability of 38.5% and O_2_ permeability of 357.8%. ZIF-302 (zeolitic imidazolate framework) particles were added into a polyimide matrix to form self-consistent MMMs [12]. MMMs exhibited a 6.05-fold increase in CO_2_ permeance compared with pure dense MMM. Liu et al. [26] described a branched polyethyleneimine (PEI) functionalized UiO-66 as the filler in a 6FDA-ODA polyimide mixed matrix membrane. The MMM with 15 wt% loading content has CO_2_/CH_4_ selectivity of 56.49.

However, when the metal-organic framework is blended with the polymer matrix, their inadequate compatibility often results in inhomogeneous dispersion [27]. Ionic liquids (ILs) are characterized by high compatibility and good thermal stability [28]. As green solvents, ionic liquids have been considered as a promising substance for CO_2_ separation. Zhang et al. [29] described a supported ionic liquid membrane (SILM) that achieved high CO_2_ permeability and selectivity for CO_2_/N_2_ (2540 Barrers and 127, respectively). A new class of CA (cellulose acetate)-derived poly(ionic liquid) as a thin film composite membrane for CO_2_ separation was reported by Nikolaeva et al. [30]. Incorporation of ionic moiety into the polymer structure resulted in a considerable threefold increase in CO_2_ permeability compared to pure CA, with only a slight decrease in selectivity.

In the present article, we first report on a simple method for preparing a series of membranes in which polyimide is capped with ionic liquid, and blended with UiO-66-NH_2_. UiO-66-NH_2_ is a metal-organic framework material composed of metal zirconium ions and 2-aminoterephthalic acid as ligands connected by metal bonds, which is often used as a filler to enhance the physicochemical properties of polymers. The amine group on UiO-66-NH_2_ particles can strengthen the affinity for CO_2_ and, hence, increase permeability. The introduction of ionic liquids helps to improve interfacial compatibility between the substrate and the MOF. Moreover, the study found that the polyimide capped by ionic liquid improved gas separation performance for the CO_2_/CH_4_ gas pair. When the ionic liquid capped polyimide was blended with MOF, permeability and separation of membranes were both enhanced. This provides a facile solution to overcome the limitation of the “trade-off.”

## 2. Materials and Methods

### 2.1. Materials

ZrCl_4_ (99%); 2-aminoterephthalic acid (NH_2_-BDC) (98%); 3,3′,4,4′-Benzophenonetetracarboxylic dianhydride (BTDA) (98%); and 4,4′-Diaminodiphenyl ether (ODA) (98%) were purchased from Aladdin (Shanghai, China). 1-carboxyethyl-3-methylimidazolium chlorine (99%) was purchased from Lanzhou Institute of Chemical Physics (Lanzhou, China). N,N-dimethylformamide (DMF) and methanol were purchased from Tianjin Kemiou Chemical Reagent Co., Ltd. (Tianjin, China).

### 2.2. Preparation of UiO-66-NH_2_

UiO-66-NH_2_ was synthesized according to a published procedure [31], with some improvements. Briefly, ZrCl_4_ (0.87 mol, 2.01 g) and 2-aminoterephthalic acid (0.87 mol, 1.56 g) were mixed with 90 mL DMF in a 250 mL flask. The mixture was heated by Microwave chemical reactor with 800 W for 30 min. The solution was centrifuged and washed with DMF and methanol to exchange solvents. The obtained solids were evaporated in a vacuum oven at 110 °C overnight [32].

### 2.3. Synthesis of PI-IL/UiO-66-NH_2_ Membranes

**Scheme 1:**
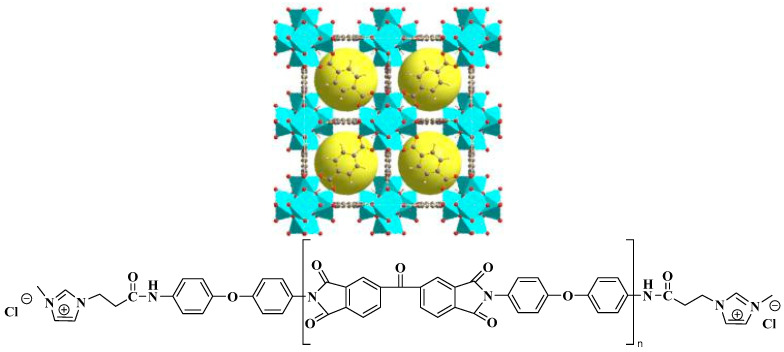
Structure of UiO-66-NH_2_ and PI-IL.

As shown in Scheme 1, PI-IL membranes were synthesized by polycondensation reaction. BTDA (1.1 mol, 1.4 g) and ODAs (1.0 mol, 0.60 g) were dissolved in DMF (3 mL) to form a polyamic acid (PAA) solution for 4 h at 20 °C. Ionic liquid 1-carboxyethyl-3-methylimidazolium hexafluorophosphate (0.10 mmol, 0.020 g) was dissolved in DMF solution, then the solution was added into polyamic acid solution. The solution was stirred for 6 h to react sufficiently. After that, UiO-66-NH_2_ (1 wt%, 2 wt%, 3 wt%, 4 wt% and 5 wt%) was dispersed in DMF and sonicated for 15 min, then the dissolved UiO-66-NH_2_ solution was mixed with the PI-IL solution and stirred for 24 h. After standing for 2 h to remove bubbles (no sedimentation of MOF due to the uniform dispersion in the casting solution [33]), the mixed solution was casted in a clean glass dish and dried at 80–280 °C for 10 h in order to obtain the mixed matrix membrane. The compositions of the synthesized membranes are listed in Table 1.

### 2.4. Characterization of the Materials

The crystal structure of the sample was determined by using X-ray diffraction (XRD) (D8 advanced diffractometer, Bruker AXS, Karlsruhe, Germany). The morphology of the MOF and cross sections of the membranes were observed by scanning electron microscope (SEM) (S-3400, Hitachi, Tokyo, Japan). Fourier Transform Infrared Spectroscopy (FT-IR) (Spectrum Two, PE company, Los Angeles, CA, USA) was used to determine the chemical structure of the samples. The nitrogen adsorption–desorption isotherm of the sample was measured with an adsorption instrument (ASAP 2020 Plus HD88, Micromeritics, Norcross, GA, USA), and the test was carried out after degassing at 120 °C for 12 h (80 mg of the sample). The particles were analyzed according to their specific surface area and pore size distribution at 77 K. A thermogravimetry analyzer (TGA 8000, Perkin Elmer, Waltham, MA, USA) was used for a thermal performance test, the flow rate of N_2_ was 40 mL/min and the heating rate was 5 °C/min.

### 2.5. Gas Permeation Measurements of Membranes

The mixed gas permeability and selectivity of the membranes were measured at 34 °C using a GTR-11MH gas permeability analyzer (GTR Tec Corporation, Uji, Kyoto). The test area was 0.785 cm^2^, the test pressure is maintained at 49 KPa, the test gas is a 1:1 mixture of CO_2_ and CH_4_, the carrier gas is H_2_ and the pressure is 0.1 MPa for testing. The gas permeability coefficient was calculated using Formula (1).

The diffusion coefficients (D) and the solubility coefficients (S) were calculated by using Equations (2) and (3) [34]:

(1) $$P=\frac{q\times K\times L}{a\times p\times t}\left(\mathrm{mL}\cdot \mathrm{cm}\cdot {\mathrm{cm}}^{-2}\cdot s^{-1}\cdot cmHg^{-1}\right)$$

(2) $$D=\frac{L^{2}}{6T}$$

(3) $$S=\frac{P}{D}$$

where *q* is the gas permeation measured by the instrument, mL; *K* is the auxiliary positive coefficient of the instrument, with a fixed value of 1.25; *L* (cm) is the thickness of the gas separation membrane; *a* is the permeation area in the instrument (set at 0.785 cm^2^); *p* (cmHg) is the pressure while the instrument is testing; and *t* (s) is the time it takes for the instrument to measure the gas separation membrane, which was determined by the specific operation of the operator.

## 3. Results

### 3.1. Fabrication and Characterization of UiO-66-NH_2_

**Figure 1:**
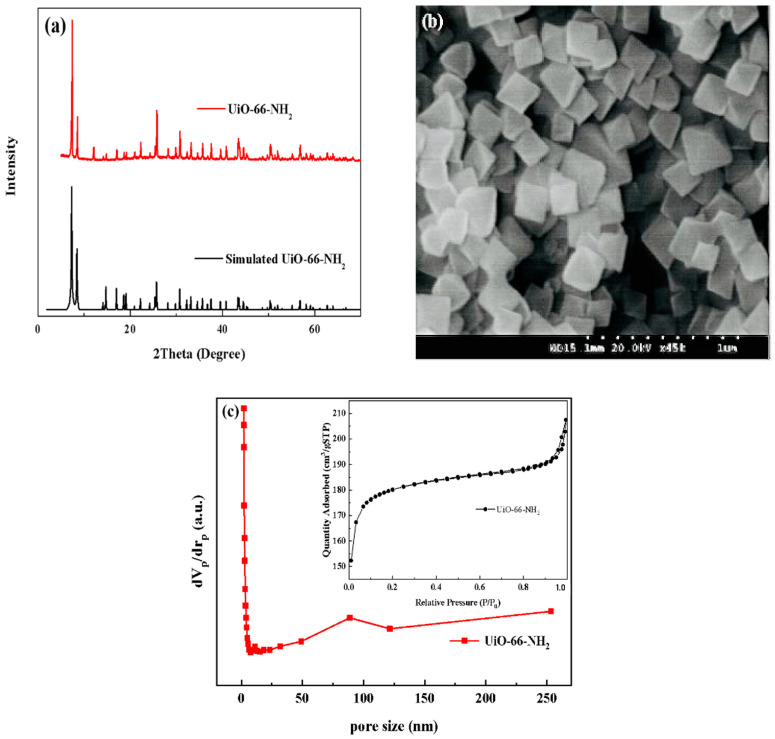
(**a**) XRD patterns of synthesized UiO-66-NH_2_, (**b**) SEM image of synthesized UiO-66-NH_2_, (**c**) nitrogen adsorption isotherm (77 K) and pore size distributions of UiO-66-NH_2_.

Microwave heating is an effective tool in organic chemistry synthesis, but it has also recently been used in the synthesis of inorganic and inorganic/organic materials. In addition, the conversion of microwave radiation to heat is often efficient and homogeneous throughout the sample, which reduces energy consumption and the necessity for heat transfer in the mixture [35]. For the reasons stated above, we employed microsynthesis technology to prepare UiO-66-NH_2_, and the reaction time decreased to 30 min, which presented a 47-fold reduction in reaction time compared to the conventional method. The yield of MOF using microwave synthesis was 50%, while the yield of MOF using traditional solvothermal process was 35%. The crystal structure of UiO-66-NH_2_ was characterized by XRD. In Figure 1a, the powder X-ray diffraction pattern of the synthesized UiO-66-NH_2_ shows excellent agreement with the simulated diffraction pattern. All of the diffraction peaks of the micro-assisted synthesized MOFs correlate well with the simulated spectrum [31,36]. Figure 1b depicts the morphology of UiO-66-NH_2_, which has a particle size of 100–200 nm and an ortho-octahedral structure, and exhibited good crystal shape regularity, particle size uniformity and crystal perfection. The nitrogen adsorption–desorption isotherm and pore size distribution curve of UiO-66-NH_2_ are presented in Figure 1c. UiO-66-NH_2_ follows Type 1 isotherm, which is indicative of microporosity. The surface area of the Brunauer–Emmett–Teller (BET) surface area was 813.25 m^2^/g, and the pore volume was 0.44 cm^3^/g, which can contribute to gas permeability. The BET area of micro-synthesized MOF is lower than that of conventional solvothermal process, while the pore is bigger. This phenomenon may be due to fewer defects; thus, the specific surface area is smaller [35]. The results of XRD, SEM and N_2_ adsorption–desorption isotherm confirmed the successful preparation of UiO-66-NH_2_ nanoparticles using microwave-assisted synthesis.

### 3.2. Characterization of MMMs

**Figure 2:**
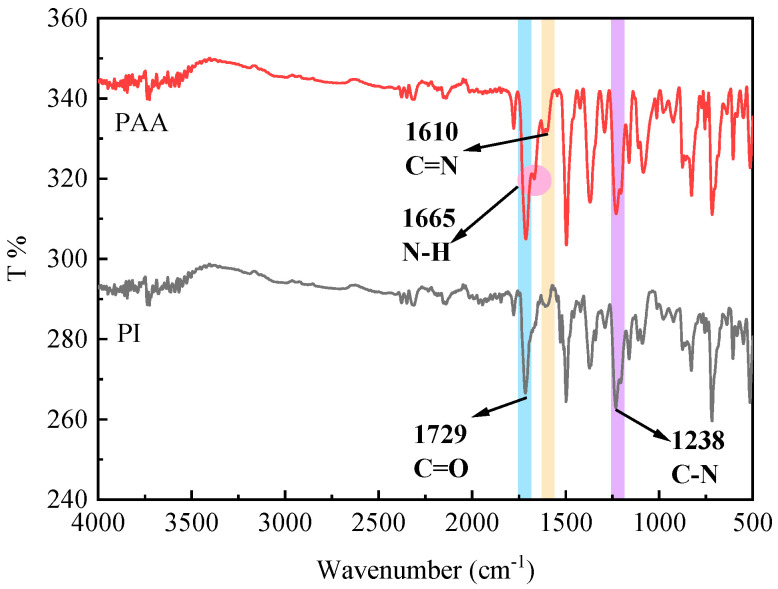
FT−IR spectrum of PAA and PI.

**Figure 3:**
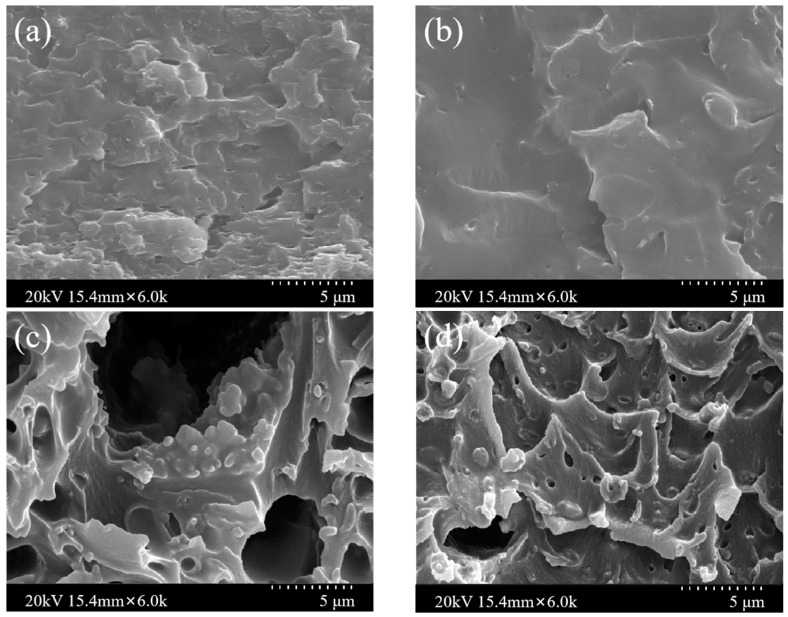

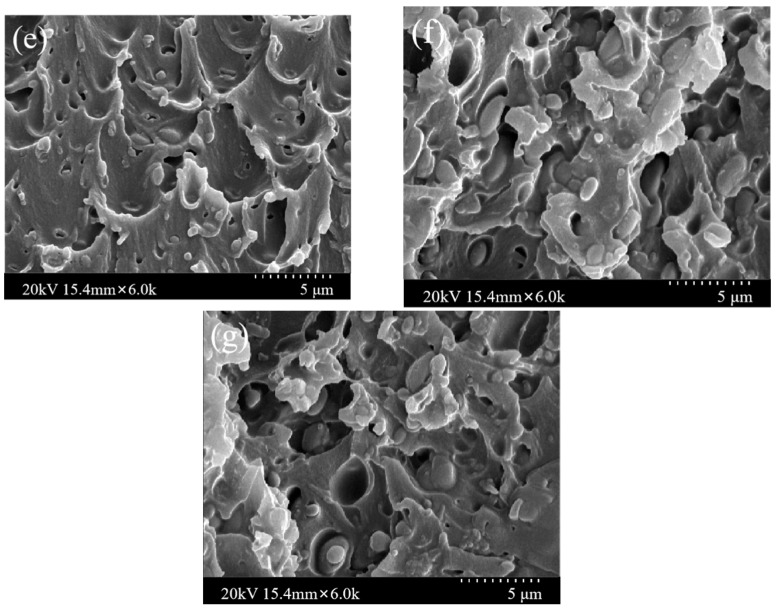
SEM images of the cross-section of pure PI (**a**), PI-IL (**b**) and PI-IL/1–5%MOF mixed matrix membranes (**c**–**g**).

In order to verify whether PI is fully imidized, the FT-IR spectra of PAA and PI are shown in Figure 2. The peak at 1665 cm^−1^ is the absorption vibration peak of –NH on the polyimide amide group, which indicated the formation of PAA. For the PI spectrum, this peak becomes weaker, indicating that PAA has been completely imidized to form PI. After imidization, the stretching vibration peak of C–N in polyimide is at 1238 cm^−1^, and the absorption peak of C=O in 1729 cm^−1^ was weakened, which proves that polyimide was formed [37]. Before SEM characterization, the membrane samples were brittled with liquid nitrogen, and the broken side was marked. As shown in Figure 3, the aggregation phase of IL is not visible in cross-section electron microscopy, which proves that the ionic liquids are not comingled in the matrix membrane but capped into polyimide. The bulky parts are the polyimide-ionic liquid matrices. The SEM images of the PI-IL/3% MOF membrane revealed that UiO-66-NH_2_ particles are well dispersed in the matrix and there is no significant agglomeration of the filler. It can be observed from Figure 3 that the number of UiO-66-NH_2_ particles in the electron micrographs increases as loading content increases. The size of the nanoparticles in the membranes is 200–300 nm, which is comparable to and slightly larger than the size of pure UiO-66-NH_2_. The particles are wrapped in a PI matrix, and the interface is blurred, demonstrating that membrane compatibility has improved. This is in accordance with the experimental theory, as the viscosity of the polyimide casting solution is very high, and the MOF particles added to it are enveloped by the casting solution. Consequently, the particle pattern in the cross-sectional electron micrographs of the film is inclined to be round, which proves that the MOF is successfully mixed into the matrix membrane. As shown in Figure S2 (in Supplementary Materials), by applying surface scanning electron microscope in mixed matrix membranes, all membranes are basically uniformly dispersed, and there was only a very small amount of agglomeration of MOF.

### 3.3. Mechanical Properties of Membranes

**Figure 4:**
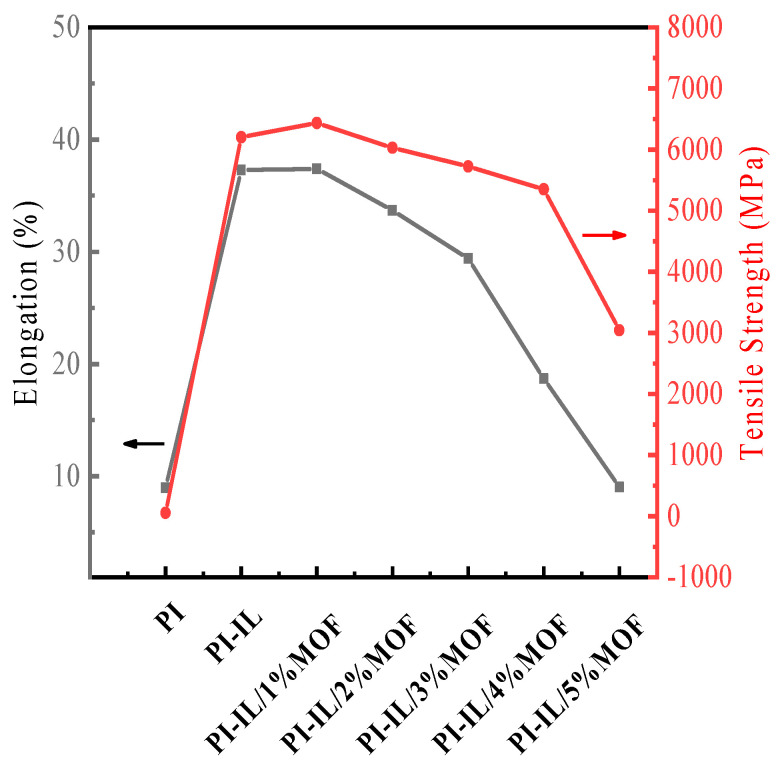
Mechanical properties of PI (PI), PI-IL (PI-IL) and PI-IL/x% (1%, 2%, 3%, 4% and 5%) MOF membranes.

As shown in Table 2, the yield strength (σs); elongation at break (εb); tensile strength (TS); and modulus of elasticity (E) of the ionic liquid-terminated polyimide membranes were measured from the tensile strength test. Figure 4 is a visualization of the trends in the mechanical properties and corresponds to the folded line point diagram of tensile strength and elongation at the break of the membranes.

The ionic liquid-capped polyimide membrane has better mechanical properties than a pure polyimide membrane, with the elongation of the break increasing from 8.96% to 37.3% and tensile strength increasing from 53.6 MPa to 6203 MPa. When IL was added to cover the end groups of the polyimide matrix, the unique electronic structure of the ionic liquid influenced the orientation of the molecular chains and increased the orientation. Simultaneously, it restricted the mobility of polyimide chain segments, increasing the degree of build up and enhancing intermolecular interactions, which resulted in its increased mechanical strength.

The tensile strength and elongation at the break of PI-IL/1% MOF reached a maximum of 37.4% and 6434 MPa, respectively. It may be due to the large interfacial interaction between the doped UiO-66-NH_2_ and the substrate polyimide. With the increase in MOF content, the tensile strength and elongation at the break of the membranes tended to decrease; this tendency may be due to the rigid structure of MOF, which makes the original excellent mechanical properties slightly inferior but still maintains good mechanical properties. This shows that all the produced mixed matrix membranes have good mechanical properties and completely fulfill the requirements under which the membranes may be used. 

### 3.4. Thermal Properties of the Membranes

**Figure 5:**
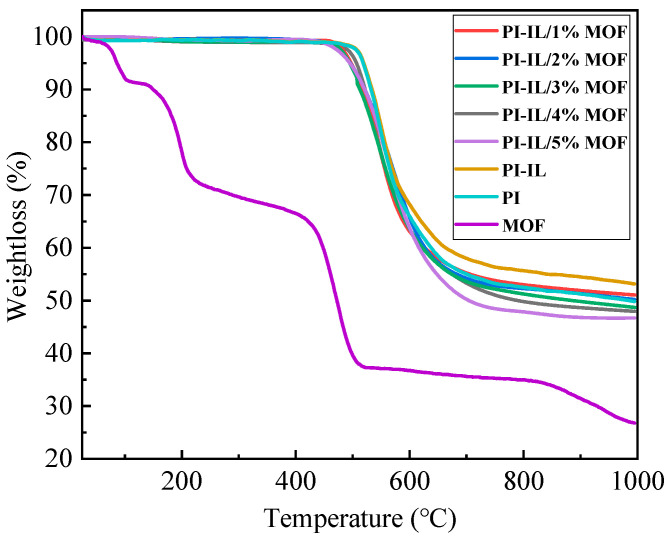
TGA curve of MOF, PI, PI-IL and PI-IL/x% MOF membranes.

A TGA measurement in a N_2_ atmosphere was used to determine the thermal stability of PI, PI-IL and PI-IL/x% MOF membranes. The TGA curve of MOF can be divided into three stages (Figure 5). As temperature figure homogeneous co-blending. The thermal decomposition mass of MOF uniformly distributed in the matrix is represented by the 5–10% mass difference. All prepared membranes can be used in normal environments and can withstand a temperature range of 0–480 °C.

### 3.5. Gas Permeation Performance of the MMMs

The prepared PI-IL/x%MOF membranes were tested for the separation performance of CO_2_ and CH_4_ mixed gas at a 1:1 ratio. Table 3 shows the gas separation performance of membranes. When PI was capped with IL, the membrane’s permeability increased to 5.19 Barrer and 74.15, which was 4.36 and 6.21-times that of pure PI membranes, respectively. The addition of ionic liquid increased CO_2_ permeability and CO_2_/CH_4_ selectivity of the PI membrane. This phenomenon was most likely due to the affinity of ionic liquid for CO_2_. The ionic liquid’s imidazole groups can interact with CO_2_ to enhance CO_2_ adsorption; consequently, the PI-IL membrane achieved higher CO_2_ permeability. It is also hypothesized that the effect of ionic liquid on gas permeability is partly due to the plasticizing effect of ionic liquids on polymers. The addition of ionic liquids to polymer membranes reduces glass transition temperature, while increasing chain mobility resulted in higher permeability [38]. Meanwhile, inorganic anions (BF_4_, NO_3_ and Cl) had a distinctive effect on its CO_2_ sorption properties. Overall, it was concluded that increasing the basicity of anions is known to enhance CO_2_ sorption [39]. The effect of IL in membranes is to increase their CO_2_ solubility while decreasing CH_4_ solubility [40]. As shown in Table 4, the solubility coefficient of the membrane increased after adding ionic liquid and MOF. In summary, the addition of IL increases the separation effect of CO_2_/CH_4_.

As shown in Table 3, introducing UiO-66-NH_2_ particles to PI-IL/x% MOF mixed matrix membranes clearly enhanced their gas permeability properties. The permeability of PI-IL/3% MOF was the highest with a value 7.61 Barrer. This behavior may be due to the addition of porous UiO-66-NH_2_ particles, which improves the transport passage of gas through the membrane and provides a higher affinity of UiO-66-NH_2_ for CO_2_ than CH_4_. The selectivity of CO_2_/CH_4_ increased to 95.10 when UiO-66-NH_2_ loading content was 3%; this could be ascribed to the molecular sieving effect of UiO-66-NH_2_ nanoparticles [41]. As the loading content increased to 4% and 5%, the selectivities dropped compared to the PI-IL/3% MOF membrane but remained higher than the pure PI membrane. These values might be attributed to a smaller degree of agglomeration of MOF particles in the matrix, blocking a small part of gas transfer. Meanwhile, the presence of MOF seems to rigidify polymeric chains; therefore, the rigidified polymeric chains around the MOF and polyimide surfaces may be one of the causes for permeability reduction. Other possibilities for the decline in selectivities include partial pore blockage of MOF by polymer chains [42]. In the case of PI-IL/1–3% MOF MMMs, the results show that the presence of IL increases the adhesion of UiO-66-NH_2_ in MMMs, since IL can also act as a wetting agent for UiO-66-NH_2_ [43]. On the other hand, IL may enhance interfacial interaction via plasticization; the presence of IL in PI-IL/1–3% MMMs improves gas separation performance.

**Figure 6:**
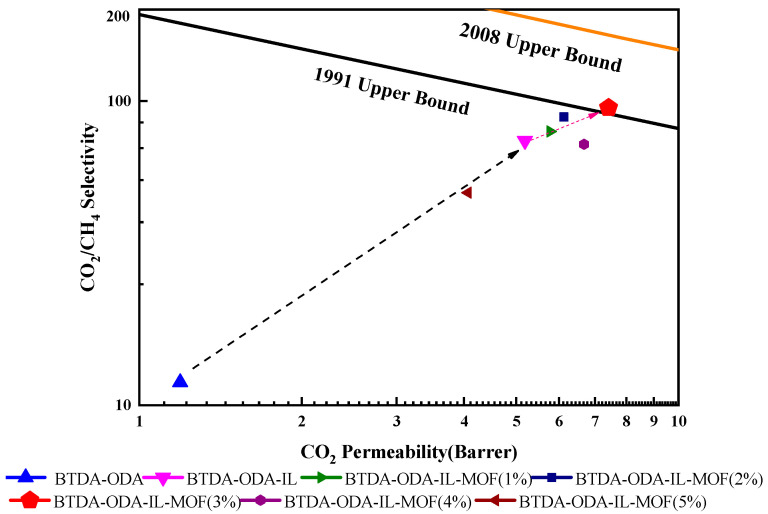
CO_2_/CH_4_ separation performance of MMMs and comparison with 1991 Robeson upper bound and 2008 Robeson upper bound.

The Robeson upper bound of PI, PI-IL and PI-IL/x% MOF membranes for the gas pair of CO_2_/CH_4_ is plotted in Figure 6. The PI-IL membrane exhibits higher CO_2_ permeability and permselectivity compared to pure PI membrane, for which its plot moves up on Robeson’s 1991 upper bound. CO_2_ and CH_4_ permselectivities of PI-IL/x% MOF mixed matrix membrane were higher than those of the PI-IL membrane, and gas separation data approaches Robeson’s 2008 upper bound when UiO-66-NH_2_ filler loading content reaches 1–2%. PI-IL filled with UiO-66-NH_2_ at 4 and 5% exhibit lower permselectivities than the PI-IL membrane, but it is still higher than the 1991 upper bound. In summary, this increase in permselectivity confirmed that the introduction of IL and UiO-66-NH_2_ can enhance gas separation performance of the fabricated membranes.

## 4. Conclusions

In conclusion, UiO-66-NH_2_ particles were used to improve CO_2_ gas permselectivity in an ionic liquid capped polyimide matrix. IL was selected to enhance the permeability of a polyimide. All the obtained mixed matrix membranes exhibited excellent mechanical and thermal properties, allowing them to meet conventional conditions of membranes. More importantly, CO_2_ permeability and selectivity of MMM exhibited an obvious improvement over PI-IL. The mixed matrix membranes capped by IL and blended with UiO-66-NH_2_ provide an efficient method to improve CO_2_ selectivity while retaining gas permeability.

## Figures and Tables

**Table 1:** Compositions of synthesized membrane.

Membrane	Composition
Pure PI	BTDA + ODA
PI-IL	BTDA + ODA + IL
PI-IL/1% MOF	1% UiO-66-NH_2_ + 99% PI-IL
PI-IL/2% MOF	2% UiO-66-NH_2_ + 98% PI-IL
PI-IL/3% MOF	3% UiO-66-NH_2_ + 97% PI-IL
PI-IL/4% MOF	4% UiO-66-NH_2_ + 96% PI-IL
PI-IL/5% MOF	5% UiO-66-NH_2_ + 95% PI-IL

**Table 2:** Mechanical properties of PI-IL/x%MOF membranes ^a^.

Sample	Yield Strength (MPa)	Elongation at Break (%)	Tensile Strength (MPa)	Modulus of Elasticity (MPa)
PI	0.61 ± 0.03	8.96 ± 0.09	53.6 ± 0.25	59.2 ± 0.21
PI-IL	54.4 ± 0.23	37.3 ± 0.16	6203 ± 2.13	177 ± 0.67
PI-IL-1% MOF	55.6 ± 0.27	37.4 ± 0.21	6434 ± 2.34	183 ± 0.78
PI-IL-2% MOF	52.4 ± 0.56	33.7 ± 0.12	6031 ± 2.54	174 ± 0.63
PI-IL-3% MOF	49.4 ± 0.41	29.4 ± 0.14	5724 ± 2.16	143 ± 0.58
PI-IL-4% MOF	46.5 ± 0.45	18.7 ± 0.15	5352 ± 2.11	105 ± 0.46
PI-IL-5% MOF	34.5 ± 0.48	9.04 ± 0.08	3042 ± 1.51	98.4 ± 0.33

Note: ^a^ Tested at 5.00 mm/min speed. The standard spline had a length of 40 mm and a width of 10 mm.

**Table 3:** CO_2_ and CH_4_ of permeability and selectivity of membranes measured at 34 °C.

No.	Sample	PCO_2_/Bar ^a^	PCH_4_/Bar ^a^	PCO_2_/PCH_4_
1	PI	1.19	0.10	11.93
2	PI-IL	5.19	0.07	74.15
3	PI-IL/1% MOF	5.57	0.07	79.53
4	PI-IL/2% MOF	6.21	0.07	88.74
5	PI-IL/3% MOF	7.61	0.08	95.10
6	PI-IL/4% MOF	6.49	0.09	72.13
7	PI-IL/5% MOF	4.01	0.08	50.08

Note: ^a^ 1 Barrer = 10^−10^ cm^3^ (STP) cm cm^−2^ s^−1^ cmHg^−1^.

**Table 4:** CO_2_ and CH_4_ solubility and diffusivity coefficients of membranes measured at 34 °C.

No.	Sample	DCO_2_ (cm^2^ s^−1^)	SCO_2_ (cm^3^ (STP) cm^2^·cmHg)	DCH_4_ (cm^2^ s^−1^)	SCH_4_ (cm^3^ (STP) cm^2^·cmHg)
1	PI	2.33 × 10^−6^	0.51 × 10^−4^	2.78 × 10^−5^	3.6 × 10^−7^
2	PI-IL	3.02 × 10^−6^	1.72 × 10^−4^	3.62 × 10^−5^	1.93 × 10^−7^
3	PI-IL/1% MOF	2.72 × 10^−6^	2.05 × 10^−4^	3.79 × 10^−5^	1.85 × 10^−7^
4	PI-IL/2% MOF	2.74 × 10^−6^	2.27 × 10^−4^	3.90 × 10^−5^	1.79 × 10^−7^
5	PI-IL/3% MOF	3.02 × 10^−6^	2.52 × 10^−4^	4.09 × 10^−5^	1.96 × 10^−7^
6	PI-IL/4% MOF	2.82 × 10^−6^	2.30 × 10^−4^	3.95 × 10^−5^	2.28 × 10^−7^
7	PI-IL/5% MOF	2.24 × 10^−6^	1.79 × 10^−4^	3.52 × 10^−5^	2.27 × 10^−7^

## Data Availability

Not applicable.

## Supplementary Materials

The following supporting information can be downloaded at: https://www.mdpi.com/article/10.3390/membranes12010034/s1, Figure S1: FT-IR spectrum of IL, PI, PI-IL; Figure S2: The surface topography of PI-IL/1%–5%MOF MMMs (a–e).
