# Supplementary material for: Optimization of a MOF Blended with Modified Polyimide Membrane for High-Performance Gas Separation
Source: Membranes (Basel). 2021 Dec 27;12(1):34. doi: 10.3390/membranes12010034 (PMC8777778; doi:10.3390/membranes12010034)
Supplement: Supplementary file 1 [file membranes-12-00034-s001.zip › membranes-1504834-supplementary.pdf]

membranes

Article

Optimization of a MOF Blended with Modified Polyimide Membrane for High-Performance Gas Separation

Yushu Zhang [1], Hongge Jia [1,*], Qingji Wang [2], Wenqiang Ma [1], Guoxing Yang [3], Shuangping Xu [1,*], Shaobin Li [1], Guiming Su [4], Yanqing Qu [1], Mingyu Zhang [1] and Pengfei Jiang [1]

1 Heilongjiang Provinces Key Laboratory of Polymeric Composite Materials, Department of Chemical and Chemical Engineering, Qiqihar University, Wenhua Street, Qiqihar, Heilongjiang Province 161006, China; 18204667530@163.com (Y.Z.); mfeather7@163.com (W.M.); qqhrlsb1022@126.com (S.L.); vipquyan-qing@163.com (Y.Q.);
zhangmingyuno1@163.com (M.Z.); jpf848185@163.com (P.J.)
2 CNPC Reasearch Institute of Safety & Environment Technology, Changping District, Beijing 102249, China; wangqingji@petroChina.com.CN
3 Synthetic Resin Laboratory Daqing Petrochemical Research Center, Petrochemical Research Institute, No. 2, Chengxiang Road, Wolitun, Longfeng District, Daqing 163714, China; ygx459@petrochina.com.cn
4 Institute of Advanced Technology, Heilongjiang Academy of Sciences, No. 52, Renhe Street, Nangang District, Harbin 150009, China; suguim@163.com
* Correspondence: jiahongge@qqhru.edu.cn (H.J.); xshp_1979_1999@163.com (S.X.)

Supplementary Materials

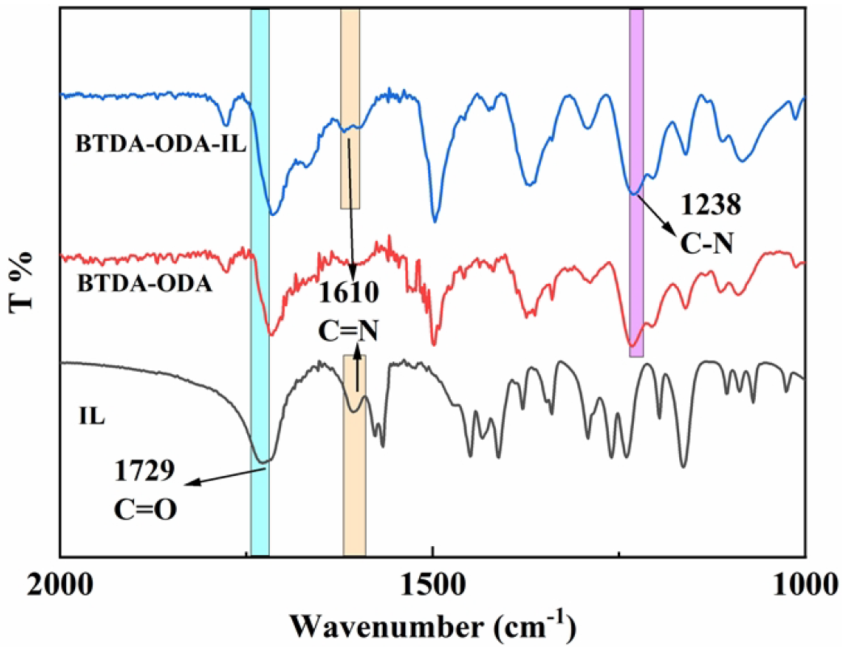

Figure S1. FT-IR spectrum of IL, PI, PI-IL. .

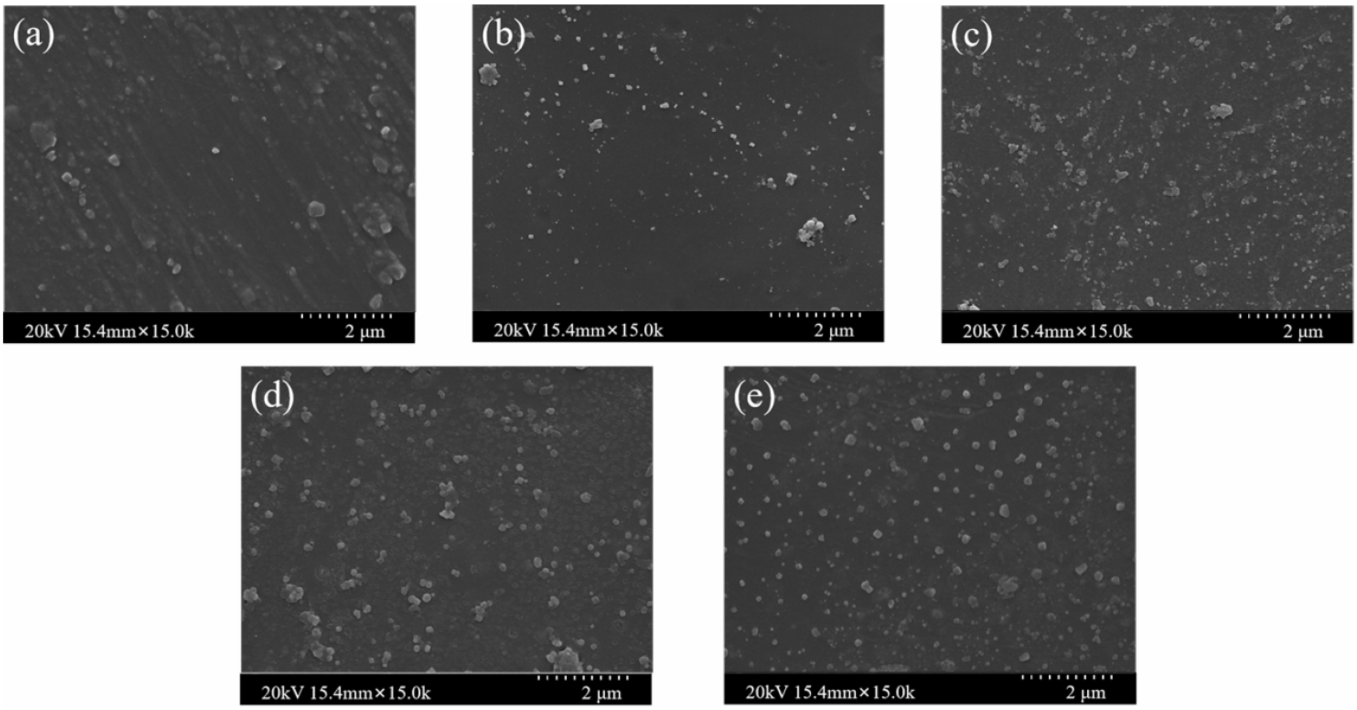

Figure S2. The surface topography of PI-IL/1%-5%MOF MMMs (a-e).
